# Prognostic effect of albumin-to-alkaline phosphatase ratio on patients with hepatocellular carcinoma: a systematic review and meta-analysis

**DOI:** 10.1038/s41598-023-28889-2

**Published:** 2023-01-31

**Authors:** Xinyuan Zhang, Yujing Xin, Yi Chen, Xiang Zhou

**Affiliations:** 1 Department of Interventional Therapy, National Cancer Centre/National Clinical Research Centre for Cancer/Cancer Hospital, Chinese Academy of Medical Sciences and Peking Union Medical College, Beijing, 100021 China; 2 Department of Interventional Radiology, First Hospital of Shanxi Medical University, Taiyuan, 030001 Shanxi China

**Keywords:** Cancer, Gastroenterology, Oncology, Risk factors

## Abstract

The prognostic value of albumin-to-alkaline phosphatase ratio (AAPR) in patients with hepatocellular carcinoma (HCC) remains controversial. This meta-analysis aims to evaluate the prognostic role of AAPR in patients with HCC. The databases of Web of Science, Embase, Cochrane Library and PubMed were comprehensively searched from inception to April 25, 2022. Pooled hazard ratio (HR) and 95% confidence interval (CI) were calculated with Stata 16.0 software for the assessment of the relationship between AAPR and overall survival (OS) as well as recurrence-free survival (RFS) in patients with HCC. A total of 2634 patients from 12 cohorts were included in this meta-analysis. The pooled results showed that lower AAPR predicted poorer OS (HR 2.02, 95% CI 1.78–2.30). Similarly, pooled results demonstrated that lower AAPR also predicted poorer RFS (HR 1.88, 95% CI 1.37–2.57). The heterogeneity for RFS by multivariate analytic results and the publication bias for OS existed, however, the subgroup analysis, meta-regression analysis as well as adjustment using trim-and-fill analysis confirmed an association between AAPR and OS as well as RFS. This meta-analysis proves that lower AAPR in patients with HCC predicted inferior survival outcomes, and AAPR might be a promising indicator for the prognosis of HCC.

## Introduction

Primary liver cancer is the sixth most commonly diagnosed cancer and the third leading cause of cancer death worldwide^1^. Hepatocellular carcinoma (HCC) accounts for 75–85% of cases of primary liver cancer^1^. Surgical resection, local thermal ablation, liver transplantation, TACE, and systemic therapy are the main treatment modalities for HCC, which have shown their efficacy in curbing overall mortality from HCC^2^. However, the survival of HCC is still poor, and projections from the World Health Organization underscore the need to improve outcomes in these patients^3^. Biomarkers have emerged as powerful tools for the diagnosis, prognosis, and prediction of treatment responses to improve patient stratification and maximize clinical benefits^3,4^. Therefore, identifying reliable and practical prognostic biomarkers before treatment administration is a highly urgent demand for HCC patients.

Increasing evidence indicates that liver function is correlated with the occurrence and progression of HCC^5–7^. Chan et al. reported that albumin (ALB) and alkaline phosphatase (ALP) had higher discrimination ability in predicting overall and disease-free survival than other parameters evaluating liver function. However, the prediction ability of albumin-to-alkaline phosphatase ratio (AAPR) calculated by dividing ALB by ALP showed the highest ability among all parameters, which exceeded that of ALB alone and ALP alone^8^. When compared with single indicator ALB or ALP, AAPR might contribute to identifying more patients with poor prognoses. In recent years, a series of studies have attempted to explore the value of AAPR as a prognostic marker in HCC^9–14^. Due to the small sample sizes, variable qualities, and inconsistent results of previous studies, a systematic summary analysis is required.

Therefore, this meta-analysis was conducted to synthetically evaluate the association between AAPR and clinical outcomes such as overall survival (OS) and recurrence-free survival (RFS) in patients with HCC based on data obtained from previous studies and provided more evidence on the clinical value of AAPR as a prognostic index for patients.

## Materials and methods

The present meta-analysis was performed according to the Preferred Reporting Items for Systematic Reviews and Meta-analyses statement (PRISM) (S1_Table)^15^.

### Search strategy

The databases of Web of Science, Embase, Cochrane Library and PubMed were comprehensively searched from inception to April 25, 2022. Search terms included "liver neoplasm", "liver cancer", “hepatocellular carcinoma", "HCC", "albumin-to-alkaline phosphatase ratio", "AAPR" and "Albumin/alkaline Phosphatase Ratio". In addition, the references of relevant studies were manually screened to identify additional potentially eligible studies. The publication language will be limited to English.

### Inclusion and exclusion criteria

The inclusion criteria of the study were as follows: (1) HCC was the only cancer diagnosis; (2) individuals with HCC were classified into two groups for AAPR cut-off value; (3) the survival endpoints were documented in the studies, including OS, RFS and disease-free recurrence (DFS); (4) the hazard ratio (HR) and corresponding 95% confidence interval (CI) for the study endpoints should be described or be calculated by sufficient data in the literature.

Exclusion criteria were as follows: (1) case reports, review articles, and comment letters; (2) insufficient data for HR and 95% CI; (3) duplicate data or analysis was identified in the studies; (3) patients were not divided into two groups for AAPR.

### Endpoints

In this meta-analysis, OS was considered as the primary endpoint, which was defined as the time from the date of the first curative operation to the date of the last follow-up, or death from any cause. RFS was considered as the second endpoint, which was defined from the time of treatment to the time of radiological evidence of tumour recurrence or metastasis. With the DFS endpoint, the relationship between AAPR and DFS was only evaluated in one study and this precluded meaningful meta-analysis.

### Data collection and quality evaluation

Two independent investigators (X.Y.Z. and Y.J.X.) evaluated and extracted all candidate articles. In case of disagreements, the two authors discussed with a third author (Y.C.). The following clinical information was extracted from the studies: the first author, year published, study region, sample size, study type, tumour stage (American Joint Committee on Cancer TNM staging system), treatment methods, cut-off value, cut-off selection, follow-up months, survival analysis and HR ratio. The HR and 95% CI in this meta-analysis were directly extracted from multivariate Cox analysis or calculated from the survival curve using Engauge Digitizer 4.1 software. The quality of all included studies was assessed by two independent authors (X.Y.Z. and Y.J.X.) using the Newcastle–Ottawa Scale (NOS)^16^. The studies with scores ≥ 6 were considered high quality.

### Statistical analysis

In this meta-analysis, the HR and 95% CI were pooled using Stata version 16.0 (Stat-Corp, College Station, TX, USA), to determine the relationship between AAPR and OS as well as RFS in patients with HCC. Cochran’s Q test combined with the I^2^ test was used to assess the statistical heterogeneity across the included cohorts. P-values < 0.1 or I^2^ ≥ 50% indicates significant heterogeneity. A random-effect model was used when substantial heterogeneity was observed; otherwise, a fixed-effect model was used. In addition, the subgroup analyses were conducted based on the year published, study region, sample size, tumour stage, cut-off value, cut-off selection, and treatment methods to investigate sources of heterogeneity. The two-sided *Z* test was performed to calculate the P-value; P < 0.05 was considered statistically significant. A sensitivity analysis was performed to assess the stability and reliability of the results of the study: (1) to assess whether the pooled results were influenced by individual studies. (2) A new analysis was conducted after excluding certain studies to evaluate the impact of these studies on the results. (3) Assess the influence of subjective judgements on outcomes. Meta-regression was conducted to detect the origin of heterogeneity. Publication bias was evaluated using the Begg's test, Egger's test, and the trim-and-fill methods^17–19^.

## Results

### Study search and characteristics of the included cohort

**Figure 1:**
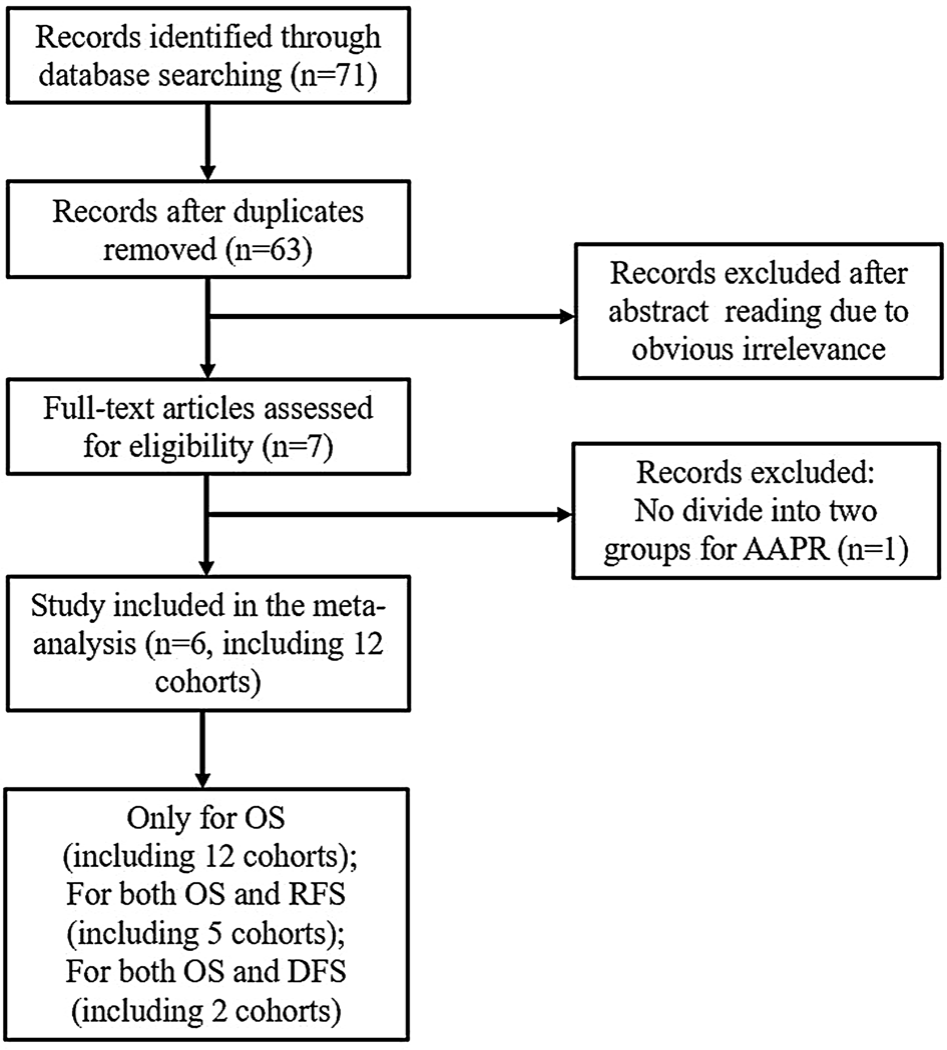
Flow diagram of the study selection procedure.

The initial search of the electronic databases yielded 71 relevant records were identified. After removing duplicate articles and reviewing the abstracts and full-text articles, only six clinical studies with 12 cohorts were ultimately incorporated in our meta-analysis^9–14^. The flow diagram for studying selection was illustrated in Fig. 1.

The main characteristics of the included studies were shown in Table 1. All included cohorts were designed retrospectively and were published between 2015 and 2021 (9 from China's mainland, the other three from Hong Kong). Sample sizes ranged from 61 to 425, with a total of 2634. Treatment methods included 6 cohorts of curative surgery (radical resection and liver transplantation) and 6 cohorts of other treatments (radiofrequency ablation, transarterial chemoembolization, systemic chemotherapy, and supportive care). The cut-off value for AAPR ranged from 0.23 to 0.44. All twelve cohorts demonstrated the association between AAPR and OS, five cohorts reported a correlation between AAPR and RFS, and only 2 cohorts reported a correlation between AAPR and DFS^10,12,13^. NOS was used to assess the quality of the included cohorts. The scores of all cohorts were ≥ 6, with the score ranging from 6 to 8 (S2_Table).

**Table 1:** Main characteristics of the included studies.

Author	Year	Region	Sample size	Study type	Tumour stage	Treatment	Cut-off value	Cut-off selection	Follow-up months	Survival analysis	HR ratio	NOS
Chan AW Training	2015	Hong Kong	217	Retrospective	I–III	Radical resection	0.23	ROC	Median 44.5	OS	Reported	8
										RFS	Reported	
Chan AW Validation 1	2015	Hong Kong	256	Retrospective	I–III	Radical resection	0.23	ROC	Median 38.9	OS	Reported	8
										RFS	Reported	
Chan AW Validation 2	2015	Hong Kong	425	Retrospective	I–IV	Palliative treatment	0.23	ROC	Median 5.3	OS	Reported	7
Cai X	2018	China	237	Retrospective	I–IV	Radical resection	0.38	ROC	NR	OS	Reported	6
Chen ZH Training	2018	China	372	Retrospective	I–IV	Transarterial chemoembolization	0.44	ROC	More than 60	OS	Reported	7
Chen ZH Validation 1	2018	China	202	Retrospective	I–IV	Supportive care	0.44	ROC	More than 60	OS	Reported	7
Chen ZH Validation 2	2018	China	82	Retrospective	I–IV	Transarterial chemoembolization	0.44	ROC	More than 60	OS	Reported	7
Li H Training	2020	China	149	Retrospective	I–III	Liver transplantation	0.38	ROC	More than 60	OS	Reported	8
										RFS	Survival curve	
Li H Validation	2020	China	61	Retrospective	I–III	Liver transplantation	0.38	ROC	More than 60	OS	Survival curve	8
										RFS	Survival curve	
Li Q	2020	China	188	Retrospective	I–IV	Radical resection	0.4	X-tile	Median 46.5	OS	Reported	7
										RFS	Reported	
Zhang F Training	2021	China	297	Retrospective	I–II	Radiofrequency ablation	0.4	X-tile	Median 28.5	OS	Reported	8
										DFS	Survival curve	
Zhang F Validation	2021	China	148	Retrospective	I–II	Radiofrequency ablation	0.4	X-tile	NR	OS	Reported	8
										DFS	Survival curve	

Palliative treatment: transarterial chemoembolization, systemic chemotherapy and supportive care; *ROC* the receiver operating characteristic, *OS* overall survival, *RFS* recurrence-free survival, *DFS* disease-free survival, *NOS* the Newcastle–Ottawa quality assessment, *NR* not reported.

### Pooled analysis of OS

**Figure 2:**
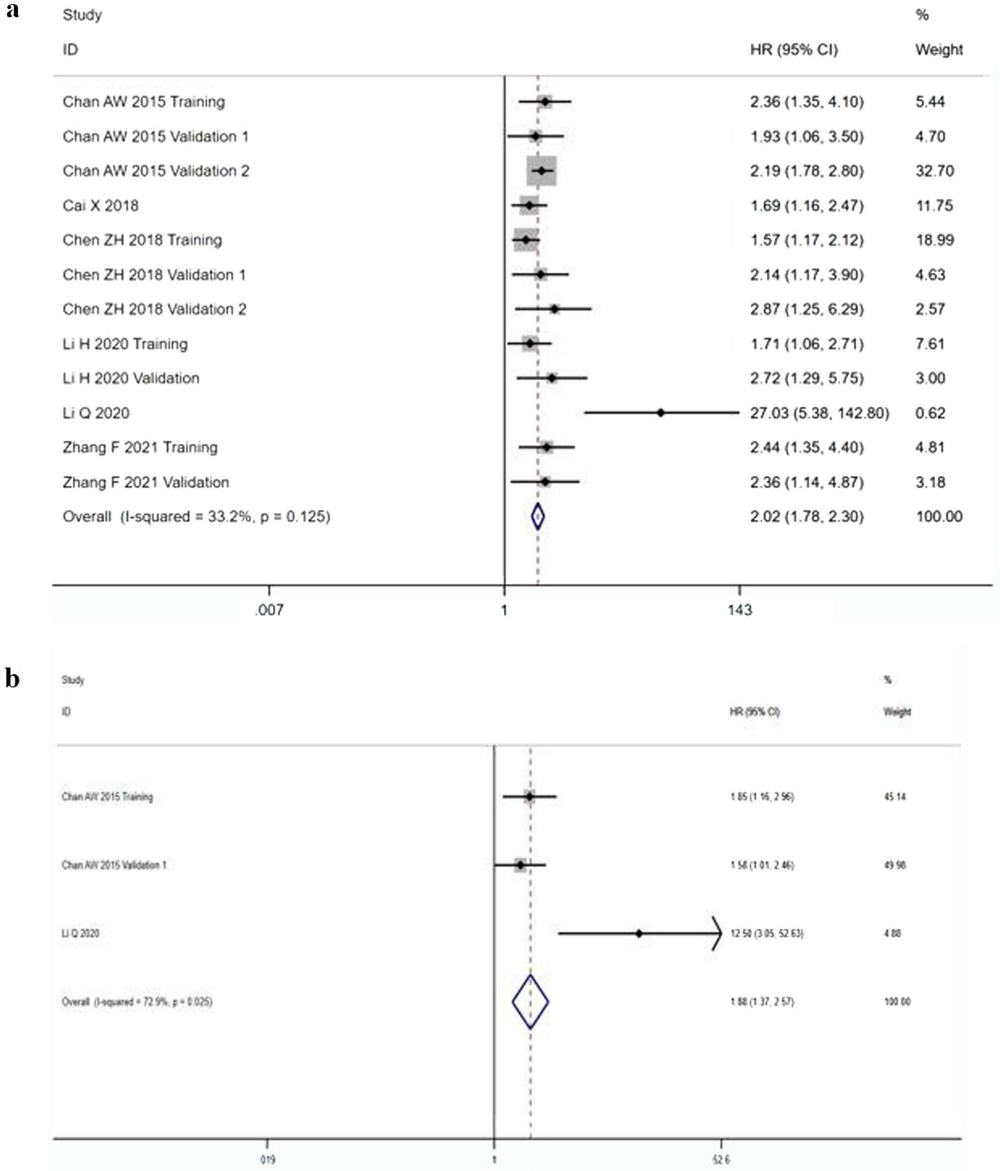
Forest plots of HR for OS (**a**) and RFS by multivariate analytic results (**b**) in patients with HCC.

A total of 2634 patients from 12 cohorts were included in the analysis of OS. Because heterogeneity between the cohorts was not statistically significant (I^2^ = 33.2%, P = 0.125), the fixed model was used for analysis. The pooled results showed that lower AAPR predicted poorer OS (HR 2.02, 95% CI 1.78–2.30) (Fig. 2a).

To explore the potential heterogeneity, two subgroups were divided from 12 cohorts providing results of OS based on the published year. The results revealed that lower AAPR predicted poorer OS in both cohorts published before 2019 (HR 1.96, CI 1.70–2.26, P < 0.001) and published after 2019 (HR 2.71, CI 1.61–4.56, P < 0.001), which demonstrated that publication year was not a source of heterogeneity and that the prognostic role of AAPR in patients with HCC may not change over time. In addition, subgroup analyses were also performed based on study region, sample sizes, tumour stage, cut-off value, cut-off selection and treatment methods. As shown in Table 2, correlations between lower AAPR and poorer OS persisted in each subgroup analysis. A meta-regression analysis was also conducted to investigate the source of heterogeneity. The results did not show a significant correlation between study region, sample size, tumour stage, cut-off value, cut-off selection or treatment methods and AAPR, demonstrating they were not the source of the heterogeneity (Table 2).

**Table 2:** Results of subgroup analysis and meta-regression analysis for OS.

Variables	No. of cohorts	No. of patients	HR (95% CI)	Heterogeneity		Z value	P value	Meta-regression P value
			Random-effects model	Ph	I^2^ (%)			
Year								0.412
Before 2019	7	1791	1.96 (1.70–2.26)	0.541	< 0.001	9.15	< 0.001	
After 2019	5	843	2.71 (1.61–4.56)	0.034	61.7	3.77	< 0.001	
Region								0.453
Hong Kong	3	898	2.18 (1.79–2.66)	0.887	< 0.001	7.72	< 0.001	
China	9	1736	2.12 (1.63–2.76)	0.054	47.7	5.62	< 0.001	
Sample size								0.385
≤ 210	6	830	2.61 (1.68–4.04)	0.057	53.5	4.30	< 0.001	
> 210	6	1804	1.95 (1.69–2.26)	0.465	< 0.001	8.96	< 0.001	
Tumour stage								0.688
No-IV	6	1128	2.14 (1.68–2.72)	0.880	< 0.001	6.17	< 0.001	
With-IV	6	1506	2.13 (1.55–2.92)	0.013	65.3	4.70	< 0.001	
Cut-off value								0.903
≤ 0.38	6	1345	2.05 (1.74–2.40)	0.740	< 0.001	8.74	< 0.001	
> 0.38	6	1289	2.48 (1.61–3.83)	0.018	63.5	4.10	< 0.001	
Cut-off selection								0.190
ROC	9	2001	1.96 (1.71–2.24)	0.637	< 0.001	9.73	< 0.001	
X-tile	3	633	3.97 (1.47–10.71)	0.021	74.2	2.72	< 0.001	
Treatment								0.985
With-curative treatment	6	1108	2.24 (1.53–3.27)	0.036	57.9	4.18	< 0.001	
No-curative treatment	6	1526	2.03 (1.74–2.38)	0.470	< 0.001	8.78	< 0.001	

*ROC* the receiver operating characteristic, *HR* hazard ratio, *95% CI* 95% confidence interval, *Ph* P-value of *Q* test for heterogeneity test.

### Pooled analysis of RFS

Regarding RFS, since the HR drawn from the survival curve is the data of univariate analysis, results were analyzed according to different sources. In total, 3 cohorts with 661 cases by multivariate analytic results were collected. The results demonstrated that lower AAPR predicted poorer RFS (HR 1.88, 95% CI 1.37–2.57), which was taken from pooled multivariate analytic results of the random-effects model (I^2^ = 72.9%, P = 0.025) (Fig. 2b). With RFS endpoint by univariate analytic results, the relationship between AAPR and RFS by univariate analytic results was only evaluated in one study and this precluded meaningful meta-analysis.

Due to the significant heterogeneity of the results by the multivariate analytic results, the subgroup analysis was then performed. The results of subgroup analyses according to the published year, study region, sample size, tumour stage, cut-off value, cut-off selection and treatment methods showed similar results in the different subgroups, demonstrating that lower AAPR predicted poorer RFS (S3_Table). A meta-regression analysis was also conducted to investigate the source of heterogeneity. The results of the meta-regression analysis suggested that the above factors were not the source of the heterogeneity.

### Sensitivity analysis and publication bias

**Figure 3:**
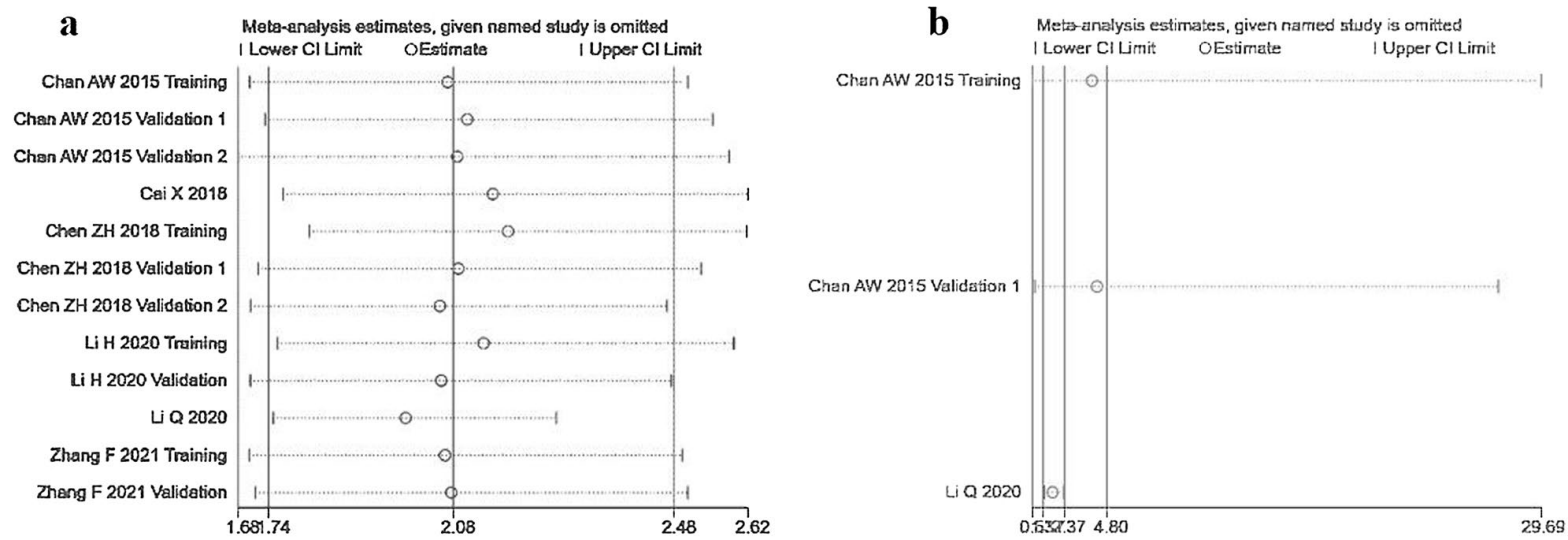
The sensitivity analysis of included cohorts. (**a**) For OS; (**b**) for RFS by multivariate analytic results.

Sensitivity analysis was performed to assess the robustness of the pooled HR with 95% CI for OS and RFS. After omitting any individual study, pooled HR was not significantly altered, indicating stable funnel plots of the meta-analysis (Fig. 3).

**Figure 4:**
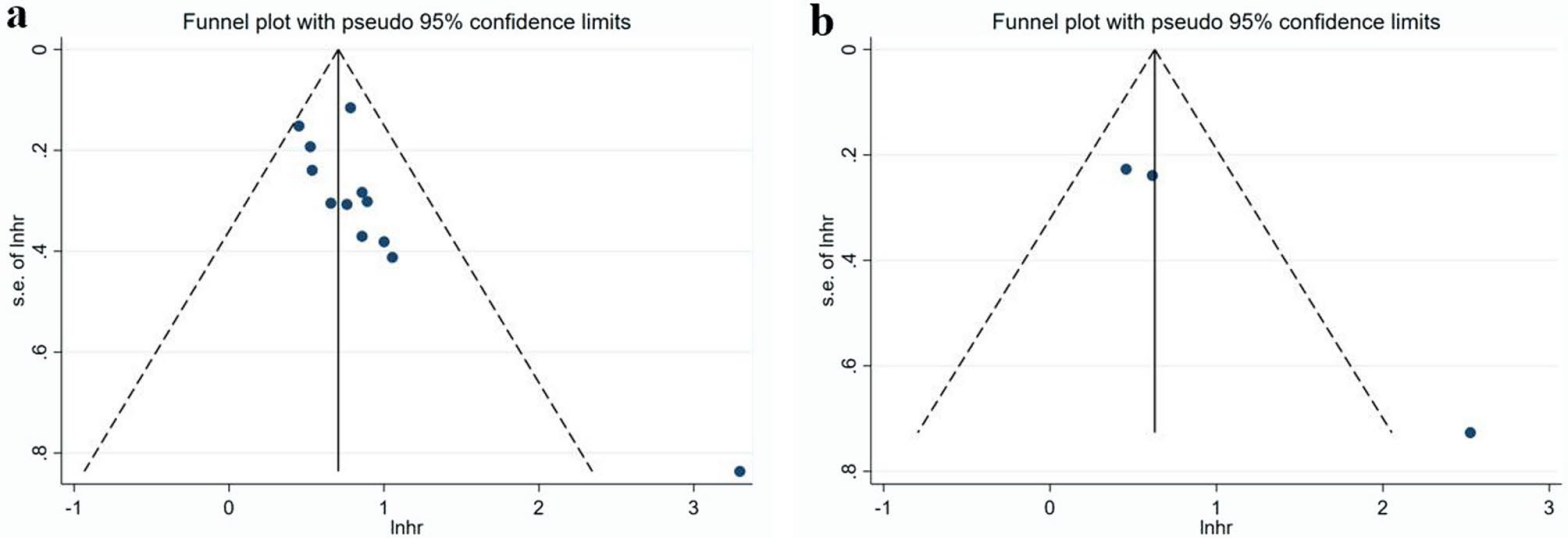
Funnel plots for the evaluation of publication bias. (**a**) For OS; (**b**) for RFS by multivariate analytic results.

**Figure 5:**
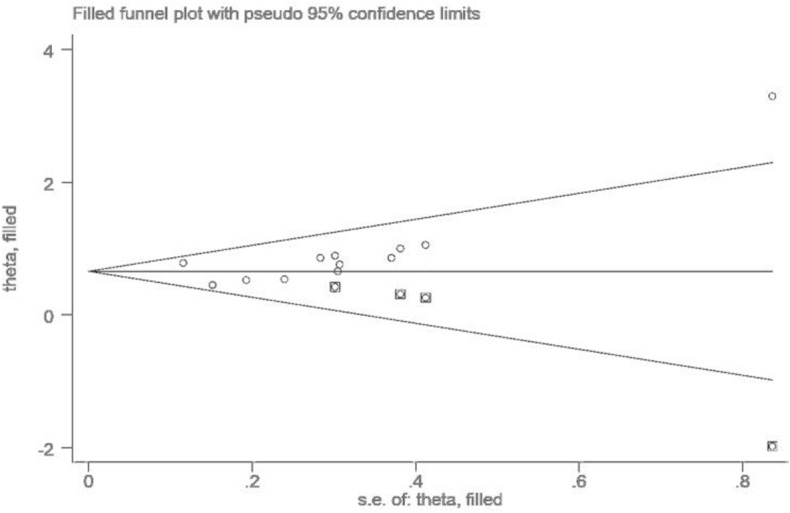
Funnel plot adjusted with trim-and-fill methods for OS.

As shown in Fig. 4, publication bias was not found in the meta-analysis with RFS by multivariate analytic results (P = 0.117, P = 0.078), which was examined by following both Begg’s and Egger’s methods. However, the publishing bias was identified in the meta-analysis with OS (P = 0.02, P = 0.04). After adjustment using trim-and-fill analysis, nonpublished cohorts were added to balance the funnel plot (Fig. 5). AAPR is still correlated with poor OS and RFS, indicating the robustness of the results.

## Discussion

It has been 7 years since the first reported study revealed that AAPR was a non-invasive indicator of prognosis in patients with HCC^10^. As a simple and composite biomarker, AAPR consisted of two routinely available biochemical and clinical parameters, ALB and ALP, which are less expensive and could be widely available. However, its prognostic value and clinical significance in patients with HCC remain unclear. To our knowledge, this current research is the first to specifically explore the relationship between AAPR and prognosis in patients with HCC.

Our pooled meta-analysis included 2634 patients with HCC from 12 cohorts. To avoid the potential bias when combining univariate and multivariate analysis data, univariate and multivariate analysis data of RFS were studied separately. The results showed that lower AAPR was associated with a poorer prognosis, including OS and RFS. Subgroup analysis of OS and RFS based on different influencing factors yielded similar results. This not only supported the conclusions of this meta-analysis but also provided some insights. The results of the subgroup analysis revealed that different years, regions, sample sizes, treatment methods and tumour stages are not factors that limited the realization of AAPR, which may explain the prognostic effect of AAPR in different regions, different stages of the HCC, and different treatment methods for patients. Thus, AAPR may be a good and promising prognostic indicator of HCC. The publication bias existed in the meta-analysis with OS, which may be because papers with positive results (studies with statistically significant results) are more likely to be accepted and published than papers with negative results (studies with non-statistically significant results). The trim-and-fill methods can estimate the number of missing studies through an iterative approach. If the pooled effect size did not change significantly, the result indicated that publication bias had little influence^19^. The trim-and-fill methods were further utilized in our study to demonstrate the association between AAPR and OS. The results showed that the results of our study were stable.

AARP is calculated by dividing the serum ALB level by the serum ALP level. ALB as a monitor of systemic inflammation reflects the protein status of the blood and the function of liver. ALB has been reported to play a role in HCC progression, which was associated with aggressive metastasis and depleting ALB significantly promoted invasion and migration of HCC^20,21^. In addition, in a clinical HCC cohort study, ALB levels were negatively correlated with tumour aggression parameters, implying that hypoalbuminemia may contribute to poor prognosis in HCC patients^22^. ALP is a hydrolase enzyme presented in all tissues and organs but mainly accumulates in the liver^23^. A previous study reported that the nuclear ALP response rate in liver cancer cell lines was higher than that in normal cells, which suggested high levels of ALP might be related to the proliferation of cancer cells^24^. Ming et al. reported that preoperative ALP level, as an independent factor for RFS and OS, could be utilized to monitor and predict recurrence in high-risk HCC patients^25^. ALP has been considered a prognostic marker in patients with HCC, which might be due to cholestasis and bile duct obstruction^26,27^. As a novel index readily derived from a simple low-cost routine blood test, AAPR may help identify more patients with poor prognosis than single-indicator decreased ALB or elevated ALP, suggesting that AAPR might be used as a more comprehensive indicator of poorer prognosis in HCC and offer more information for clinicians including tumour burden, inflammation status, and nutrition status^11^. The optimal cut-off for the AAPR was determined by the ROC curve according to overall and disease-free survival. All patients could be divided into two groups based on their AAPR cut-off value: high-risk AAPR group and low-risk AAPR group, which allows for risk stratification of patients to aid clinicians in following disease progression and help physicians make appropriate clinical decisions.

We followed PRISM guidelines strictly to perform this meta-analysis, but our study still had several limitations. First, as all included studies in this analysis were retrospective designs, selection biases could not be avoided. Second, only papers published in English have been included in the current study. Thus, we may have missed data from studies published in other languages. Third, the lack of publication of negative results in data analysis could lead to an overestimation of the value of AAPR. Fourth, the number of included studies was limited and more large-sample size studies are needed to fully confirm the relationship between AAPR and HCC prognosis.

## Conclusion

In conclusion, this meta-analysis proves that lower AAPR in patients with HCC predicted inferior survival outcomes. AAPR might be a promising indicator for the prognosis of HCC. The conclusion needs to be verified by further prospective cohort studies with larger sample sizes and a more rigorous design.

## Supplementary Information

Supplementary Tables.

## Data Availability

The datasets used and/or analysed during the current study are available from the corresponding author on reasonable request.
